# Association between biological aging and periodontitis using NHANES 2009–2014 and mendelian randomization

**DOI:** 10.1038/s41598-024-61002-9

**Published:** 2024-05-02

**Authors:** Sihong Li, Chang Wen, Xueying Bai, Dong Yang

**Affiliations:** 1 State Key Laboratory of Oral and Maxillofacial Reconstruction and Regeneration, Key Laboratory of Oral Biomedicine Ministry of Education, Hubei Key Laboratory of Stomatology, School and Hospital of Stomatology, Wuhan University, Wuhan, China; 2 Department of Periodontology, School and Hospital of Stomatology, Wuhan University, 237 Luoyu Road, Hongshan District, Wuhan, 430079 China

**Keywords:** Aging, Periodontitis, NHANES, Mendelian randomization analysis, Diseases, Health care, Medical research, Risk factors

## Abstract

Aging is a recognized risk factor for periodontitis, while biological aging could provide more accurate insights into an individual's functional status. This study aimed to investigate the potential association between biological aging and periodontitis. Epidemiological data from 9803 participants in the 2009–2014 National Health and Nutrition Examination Survey were analyzed at a cross-sectional level to assess this link. Three biological ages [Klemera–Doubal method (KDM), PhenoAge, and homeostatic dysregulation (HD)] and two measures of accelerated biological aging (BioAgeAccel and PhenoAgeAccel) were set as primary exposure and were calculated. Logistic regression and restricted cubic spline regression were employed to examine the relationship between biological aging and periodontitis. Additionally, Mendelian randomization analysis was conducted to explore the causal connection between accelerated biological aging and periodontitis. After adjusting for age, gender, race, educational level, marital status, ratio of family income, and disease conditions, this study, found a significant association between subjects with older higher biological ages, accelerated biological aging, and periodontitis. Specifically, for a per year increase in the three biological ages (HD, KDM, and PhenoAge), the risk of periodontitis increases by 15%, 3%, and 4% respectively. Individuals who had positive BioAgeAccel or PhenoAgeAccel were 20% or 37% more likely to develop periodontitis compared with those who had negative BioAgeAccel or PhenoAgeAccel. Furthermore, a significant non-linear positive relationship was observed between the three biological ages, accelerated biological aging, and periodontitis. However, the Mendelian randomization analysis indicated no causal effect of accelerated biological aging on periodontitis. Our findings suggest that biological aging may contribute to the risk of periodontitis, highlighting the potential utility of preventive strategies targeting aging-related pathways in reducing periodontitis risk among older adults.

## Introduction

Periodontitis poses a significant global burden in an aging world. Approximately 90% of adults worldwide are affected by periodontitis, which can lead to severe cases of tooth loss, impairing masticatory ability, aesthetics, and overall quality of life, particularly among the elderly population^1^. Biological aging is a natural process characterized by the gradual decline in the functional capacity of various organs and systems^2^. It is commonly believed that older individuals are more susceptible to periodontitis. However, since individuals age at different rates despite having the same chronological age, biological age better represents an individual's functional status^3^. Therefore, it is imperative to investigate the impact of biological aging on periodontitis.

Several traditional estimators of biological aging have been utilized previously for periodontal health. However, the relationship between the development of periodontitis and these accelerated biological aging estimators has been less studied. For example, telomere, repetitive non-coding DNA sequence found at the end of chromosomes, whose length gradually shortened with each cell division until cells underwent aging and apoptosis, was considered a marker of aging^4^. A recent cross-sectional study utilizing telomere length as a measure of aging revealed an association between periodontitis and telomere shortening^5^. Nevertheless, a composite clinical biomarker may serve as a stronger representation of biological aging than telomeres^6^.

In a cohort study involving 2049 participants, a biological age score was proposed using multiple composite biomarkers, accurately predicting tooth loss over 10 years^7^. The advanced aging estimator was developed based on six clinical biomarkers: waist circumference, low-density lipoprotein cholesterol, fibrinogen, blood pressure, white blood cell count, and glycated hemoglobin. This study demonstrated that individuals with a younger biological age had fewer periodontal risk factors, even if their chronological age was the same^8^. However, the biomarkers included in this study are limited and not fully representative. Research on aging-related biomarkers has a long-standing history and is an ongoing field of study today^9^. Furthermore, existing epidemiological studies investigating the association between biological aging and periodontitis are scarce, with small sample sizes, and inadequate control for key confounding variables^10,11^.

Representative biological aging measurements based on composite clinical biomarkers have been proposed and validated, including homeostatic dysregulation (HD), Klemera–Doubal (KDM), and the PhenoAge method according to the previous studies^12,13^. These three biological ages, which incorporate biomarkers from multiple organ systems, consistently correlate well with chronological age^14^. All algorithms are based on NHANES III (1988–1994) using the R package "BioAge". They not only demonstrate the ability to accurately predict aging-related morbidity and mortality^3^ but also exhibit sensitivity to various causes that are hypothesized to accelerate aging^15,16^. Accelerated biological aging, quantified as the residual when regressing biological age on chronological age, serves as a measure of the speed of biological aging. However, it remains unclear whether the three biological ages (HD, KDM, and PhenoAge) and two accelerated biological aging measures (BioAgeAccel and PhenoAgeAccel) are associated with periodontitis.

Therefore, the present study aims to investigate the association between biological ages, accelerated biological aging, and periodontitis using epidemiological data. Considering the undetermined association^17^, we intend to assess the causal relationship using Mendelian randomization (MR) studies.

## Methods

**Figure 1:**
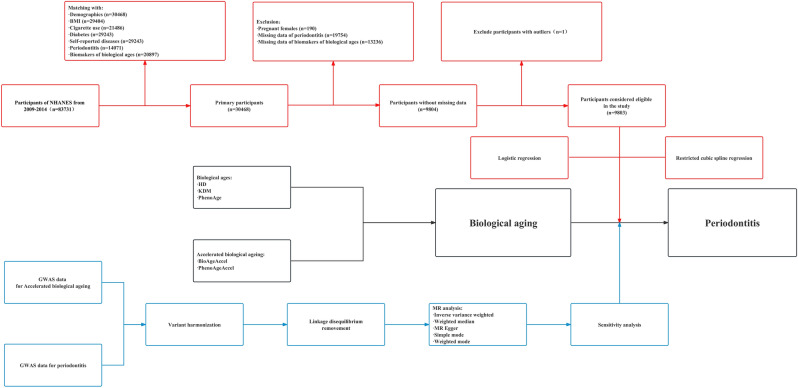
Flowchart of study design. The red represented the cross-sectional study, with biological ages and accelerated biological aging as independent variables and periodontitis as dependent variables. The blue represented the MR analyses, with accelerated biological aging as exposure and periodontitis as the outcome.

Figure 1 illustrates the study design procedures, which involved correlation analysis using the National Health and Nutrition Examination Survey (NHANES) database (https://www.cdc.gov/nchs/nhanes/index.htm)^18^ and causal inference using two-sample MR. For participants with missing values in the NHANES study, we performed a deletion process. Outliers were defined as anomalous observations that were far from the other values and were likewise removed.

### Cross-sectional study

#### Participants in NHANES

This study utilized publicly available data from NHANES. A total of 83,731 participants from the 2009–2014 cohort of NHANES were included. To investigate the association between biological aging and periodontitis, we matched data from participants aged 20 years and older who underwent periodontal examination and had valid biological aging measures (9,803 participants).

#### Biological aging assessment in NHANES

Three different measures were used to assess biological aging: the Klemera–Doubal method (KDM), PhenoAge, and homeostatic dysregulation (HD)^19^.

KDM^20^ is derived from a regression analysis of various aging-related biomarkers on chronological age in a reference population. Comparing an individual's KDM to their chronological age provides insights into their health status and risk for age-related diseases. An elevated KDM compared to chronological age suggests an increased susceptibility to age-related illnesses, disability, and mortality, while a lower KDM indicates healthier aging. PhenoAge is calculated from a multivariate analysis of mortality hazards and uses a set of nine clinical biomarkers to predict an individual's age. It provides a more accurate assessment of an individual's rate of aging and risk of age-related diseases compared to chronological age alone^21^. HD^22^ is calculated based on a set of biomarkers unrelated to chronological age. The Mahalanobis distance is used to quantify the difference between an individual's biomarker profile and a "healthy" reference sample, with higher values indicating greater deviation from the reference sample. High HD values indicate advanced biological aging and increased risk of disease and mortality, while low HD values indicate delayed aging and reduced risk^19^.

KDM, PhenoAge, and HD were computed using the R package "BioAge." All algorithms were trained on 11 biomarkers from NHANES III (1988–1994): albumin, alkaline phosphatase, blood urea nitrogen, creatinine, uric acid, lymphocyte percentage, mean cell volume, white blood cell count, systolic blood pressure, total cholesterol, and hemoglobin A1C. These variables for all age assessments are all available in NHANES 2009–2014.

To measure the speed of aging, BioAgeAccel and PhenoAgeAccel were calculated as indicators of accelerated biological aging. BioAgeAccel is defined as the residual from a linear regression of KDM on chronological age. Similarly, PhenoAgeAccel is the residual from a linear regression of PhenoAge on chronological age. A positive value indicates accelerated aging, while a negative value suggests the opposite^23^.

#### Periodontal diagnosis in NHANES

Periodontitis was diagnosed based on the measurement of periodontal pocket probing depth (PD) and attachment loss (AL). According to CDC criteria^24^, the diagnosis is as follows:

Mild periodontitis was defined as having two or more interproximal sites with AL ≥ 3 mm and two or more interproximal sites with PD ≥ 4 mm (not on the same tooth), or one interproximal site with PD ≥ 5 mm.

Moderate periodontitis was diagnosed when two or more interproximal sites have AL ≥ 4 mm or two or more interproximal sites had PD ≥ 5 mm, but not on the same tooth.

Severe periodontitis was diagnosed when two or more interproximal sites had AL ≥ 6 mm (not on the same tooth), and at least one interproximal site had PD ≥ 5 mm.

If none of these conditions were met, the patient was diagnosed with "no periodontitis".

#### Covariates in NHANES

Demographic data, including age, sex (male, female), race (Mexican American, Other Hispanic, Non-Hispanic White, Non-Hispanic Black, Other Race), education (junior high school or below, high school, college or above), marital status (married, never married, others), and ratio of family income to poverty, were collected through person demographics questionnaires and considered in this analysis. Smoking status and self-reported diseases (including diabetes, arthritis, liver disease, and cancer) were obtained through interviews with trained interviewers and also included for analyses. Never smoking was defined as smoking less than 100 cigarettes in a lifetime. A former smoker smoked more than 100 cigarettes in a lifetime but had quit. The current smoker smoked more than 100 cigarettes in life and currently smokes. Disease status was determined based on patient-reported medical history. In addition, height and weight were considered and measured at a specialized Mobile Examination Center to calculate body mass index (BMI). All of the data above are available in the NHANES database (https://www.cdc.gov/nchs/nhanes/index.htm).

#### Statistical analysis

All analyses were performed using R (version 4.2.2, www.R-project.org). Six-year survey weights were calculated according to NHANES analytical guidelines to generate nationally representative estimates. Descriptive statistics were used to present the mean and standard error of continuous variables, as well as the count and weighted percentages of categorical variables. T-tests and chi-square tests were conducted to assess the associations between periodontitis and continuous and categorical variables. Multiple logistic regression and cubic spline regression were used to assess the association between periodontitis and the three biological aging measures and two age accelerations, with adjustments for covariates. If the association between the two was nonlinear, the cutoff value was calculated to determine the range of the strongest association between the independent variable and the dependent variable. The significance level was set at 5%. Cubic spline regression in our study was a regression method used to fit data points, approximating a given data point by constructing a piecewise cubic polynomial function. Within each segmented interval, cubic spline regression used a cubic polynomial function to represent the trend of data changes. These polynomial functions had continuous first and second derivatives at the connection points (referred to as nodes) in each interval, ensuring the smoothness of the entire regression curve.

### Mendelian randomization

#### Data source

For the two age accelerations, we used the latest and only Genome-wide association study (GWAS) research data, which estimated clinical biochemistry markers in 379,703 European-descent participants from the UK Biobank^25^. The periodontitis-related GWAS summary data, including 17,353 periodontitis cases and 28,210 controls, were obtained from the GLIDE consortium^26^. All participants were of European ancestry.

#### Selection of genetic instruments

MR analyses require satisfying three criteria: (1) relevance assumption: the genetic variants must be correlated with accelerated biological aging; (2) independence assumption: the genetic variants should not be affected by confounding factors; and (3) exclusion restriction assumption: the genetic variants should affect periodontitis only through accelerated biological aging.

To identify the causal relationship between the acceleration of biological aging and periodontitis, we used two sets of genetic instruments representing different calculations of accelerated biological aging: BioAgeAccel and PhenoAgeAccel. Accelerated aging refers to being older than one's chronological age^10^. We selected 995 and 7,561 single nucleotide polymorphisms (SNPs), respectively, that were correlated with BioAgeAccel and PhenoAgeAccel (*p* < 5 × 10^−8^) as instrumental variables (IVs). Then, to ensure the independence of the IVs, we further selected 21 and 59 SNPs, respectively, with coefficients of linkage disequilibrium < 0.001. The SNPs used as IVs in this study are listed in Tables S1 and S2. Subsequently, we removed periodontitis-related SNPs with a threshold of 5 × 10^− 8^ to ensure that the genetic instruments satisfied the exclusion restriction assumption.

#### MR analyses

We employed the inverse-variance-weighted (IVW) method as our primary analysis method. IVW combines the estimated values of each genetic variant into a weighted average, where the weight is inversely proportional to the variance of the genetic variation estimate^27^. Because IVW assumes that all genetic variants are valid, it maximizes statistical power in MR analysis and is susceptible to pleiotropic bias^28^. Therefore, we also performed MR Egger, weighted median, simple mode, and weighted mode analyses, which allow for some degree of selection bias and assume a certain degree of heterogeneity^29,30^, to collectively analyze the causal effects of accelerated biological aging on periodontitis.

The Cochran's Q test, MR-Egger intercept test, and MR-PRESSO global test were used to identify potential heterogeneity and directional pleiotropy. A *p*-value > 0.05 for Cochran's Q test^31^ indicates no heterogeneity, while a *p*-value > 0.05 for the MR-PRESSO global test and MR-Egger intercept test^32^ indicates no pleiotropy. Additionally, we conducted a leave-one-SNP-out analysis to assess the influence of individual variants on the overall causal associations.

### Ethics declarations

Data collection of NHANES was approved by the NCHS Research Ethics Review Board (ERB) and the data are publicly available. Data used in MR analysis are publicly available and ethically approved, and the subjects have given their informed consent.

## Results

### Baseline characteristics of NHANES participants

A total of 9803 study participants aged 20 and older who had data on biological ages and periodontitis were included in the analysis. Table 1 presents the general characteristics of the participants, including demographic factors, periodontitis-related diseases, biological ages, and periodontitis status. Among the participants, 5015 (51.16%) were classified as having periodontitis. The weighted mean age of the included participants, consisting of 4916 males and 4887 females, was 50.97 ± 0.25 years. Furthermore, 37.37% of the participants had a BMI greater than or equal to 30, 69.47% were non-Hispanic white, 64.04% had a college degree or higher, and 63.63% were married. Significant differences were observed between individuals with periodontitis and those without periodontitis in terms of age, gender, race, education level, marital status, ratio of family income to poverty, cigarette use, BMI, diabetes, and arthritis. Moreover, participants with periodontitis exhibited higher biological ages compared to those with periodontal health (HD:2.19 vs 2.03, KDM: 52.9 vs 45.8, PhenoAge: 52.9 vs 44.7).

**Table 1:** Baseline characteristics of the NHANES participants.

Characteristics	Overall	Periodontitis	Control	*p*
N	9803	5015	4788	
Age, years, mean (SE)	51.0 (0.2)	54.7 (0.4)	48.3 (0.3)	< 0.001
Biological ages				
HD, mean (SE)	2.10 (0.01)	2.19 (0.02)	2.03 (0.01)	< 0.001
KDM, mean (SE)	48.8 (0.3)	52.9 (0.4)	45.8 (0.3)	< 0.001
PhenoAge, mean (SE)	48.2 (0.3)	52.9 (0.4)	44.7 (0.3)	< 0.001
Gender, N (%)				
Male	4916 (49.9)	2948 (59.0)	1968 (43.3)	< 0.001
Female	4887(50.1)	2067 (41.0)	2820 (56.7)	
Race, N (%)				
Mexican American	1425 (8.1)	907 (11.5)	518 (5.7)	< 0.001
Other Hispanic	977 (5.3)	513 (6.2)	464 (4.7)	
Non-Hispanic White	4306 (69.5)	1849 (60.9)	2457 (75.7)	
Non-Hispanic Black	1946 (10.1)	1200 (13.7)	746 (7.4)	
Other race	1149 (7.0)	546 (7.7)	603 (6.5)	
Education level, N (%)				
Junior high school or below	2260 (15.1)	1567 (23.2)	693 (9.2)	< 0.001
High school	2121 (20.9)	1263 (26.1)	858 (17.1)	
College or above	5409 (64.0)	2176 (50.7)	3233 (73.7)	
Marital status, N (%)				
Married	5748 (63.6)	2742 (56.9)	3006 (68.5)	< 0.001
Never married	1101 (10.1)	547 (10.8)	554 (9.5)	
Others	2948 (26.3)	1721 (32.3)	1227 (22.0)	
Ratio of family income to poverty, N (%)				
< 1	1707 (11.6)	1102 (17.0)	605 (7.7)	< 0.001
≥ 1	7306 (88.4)	3462 (83.0)	3844 (92.3)	
Cigarette use, N (%)				
Never	5477 (56.1)	2405 (45.8)	3072 (63.6)	< 0.001
Former	2482 (26.6)	1370 (28.5)	1112 (25.1)	
Current	1839 (17.3)	1235 (25.7)	604 (11.3)	
BMI, N (%)				
Normal or low-weight	2619 (26.8)	1275 (24.9)	1344 (28.5)	0.001
Overweight	3382 (34.7)	1729 (35.5)	1653 (35.8)	
Obese	3753 (38.5)	1982 (39.6)	1771 (35.7)	
Diabetes, N (%)				
Non-diabetes	7836 (80.0)	3817 (76.1)	4019 (86.2)	< 0.001
Pre-diabetes	744 (7.6)	375 (7.5)	369 (7.2)	
Diabetes	1218 (12.4)	821 (16.4)	397 (6.6)	
Arthritis, N (%)				
Yes	2633 (25.8)	1488 (28.8)	1145 (23.7)	< 0.001
No	7149 (74.2)	3515 (71.2)	3634 (76.3)	
Liver disease, N (%)				
Yes	371 (3.3)	203 (3.6)	168 (3.1)	0.344
No	9414 (96.7)	4797 (96.4)	4617 (96.9)	
Cancer, N (%)				
Yes	908 (10.5)	499 (11.3)	409 (10.0)	0.183
No	8887 (89.5)	4509 (88.7)	4378 (90.)	

Continuous variables were presented as mean (SE); Categorical variables were presented as N (%); means (SE) and % were weight-adjusted. KDM: Klemera–Doubal method; HD: homeostatic dysregulation; BMI: body mass index.

### Logistic regression analysis of the association between biological aging and periodontitis

Table 2 presents the associations of periodontitis with biological ages. After adjusting for age, gender, and race (model 1), we found a significant association between older biological ages and accelerated biological aging with periodontitis. This finding remained consistent across multinomial logistic regression models that included additional adjustments for educational level, marital status, and ratio of family income (model 2). Further adjustment for disease conditions in model 3 did not considerably affect the results. In model 3, for per year increase in the three biological ages (HD, KDM, and PhenoAge), the risk of periodontitis increases by 15% (OR 1.15, 95% CI 1.08–1.22, *p* < 0.001), 3% (OR 1.03, 95% CI 1.02–1.03), and 4% (OR 1.04, 95% CI 1.03–1.06, *p* < 0.001) respectively. Biological aging acceleration defined as the residual from regressing biological age on chronological age, including BioAgeAccel and PhenoAgeAccel, was also related to periodontitis. Biological aging acceleration indicates older biological ages. Individuals who had positive value on BioAgeAccel or PhenoAgeAccel were 20% (OR 1.20, 95% CI 1.09–1.32, *p* < 0.001) or 37% (OR 1.37, 95% CI 1.24–1.52, *p* < 0.001) more likely to develop periodontitis compared with those who had negative BioAgeAccel or PhenoAgeAccel.

**Table 2:** Associations of biological ages and accelerated biological aging with periodontitis.

Biological aging	Model 1			Model 2		Model 3	
	OR (95% CI)	*p*	OR (95% CI)		*p*	OR (95% CI)	*p*
HD	1.27 (1.21,1.34)	< 0.001	1.17 (1.10,1.23)		< 0.001	1.15 (1.08,1.22)	< 0.001
KDM	1.04 (1.03, 1.05)	< 0.001	1.03 (1.02,1.04)		< 0.001	1.03 (1.02,1.03)	< 0.001
PhenoAge	1.07 (1.06,1.07)	< 0.001	1.05 (1.03,1.06)		< 0.001	1.04 (1.03,1.06)	< 0.001
BioAgeAccel	1.35 (1.24, 1.47)	< 0.001	1.22 (1.11, 1.34)		< 0.001	1.20 (1.09, 1.32)	< 0.001
PhenoAgeAccel	1.64 (1.51, 1.79)	< 0.001	1.40 (1.27, 1.54)		< 0.001	1.37 (1.24, 1.52)	< 0.001

Model 1: Adjusting for age, gender, race;

Model 2: Model 1 + adjusted for educational level, marital status, ratio of family income to poverty, BMI, and cigarette use.

Model 3: Model 2 + adjusted for diabetes, arthritis, liver disease, and cancer.

HD: homeostatic dysregulation; KDM: Klemera–Doubal method; OR: Odds ratio; CI: Confidence interval;

### Restricted cubic spline regression analysis of the association between biological aging and periodontitis

**Figure 2:**
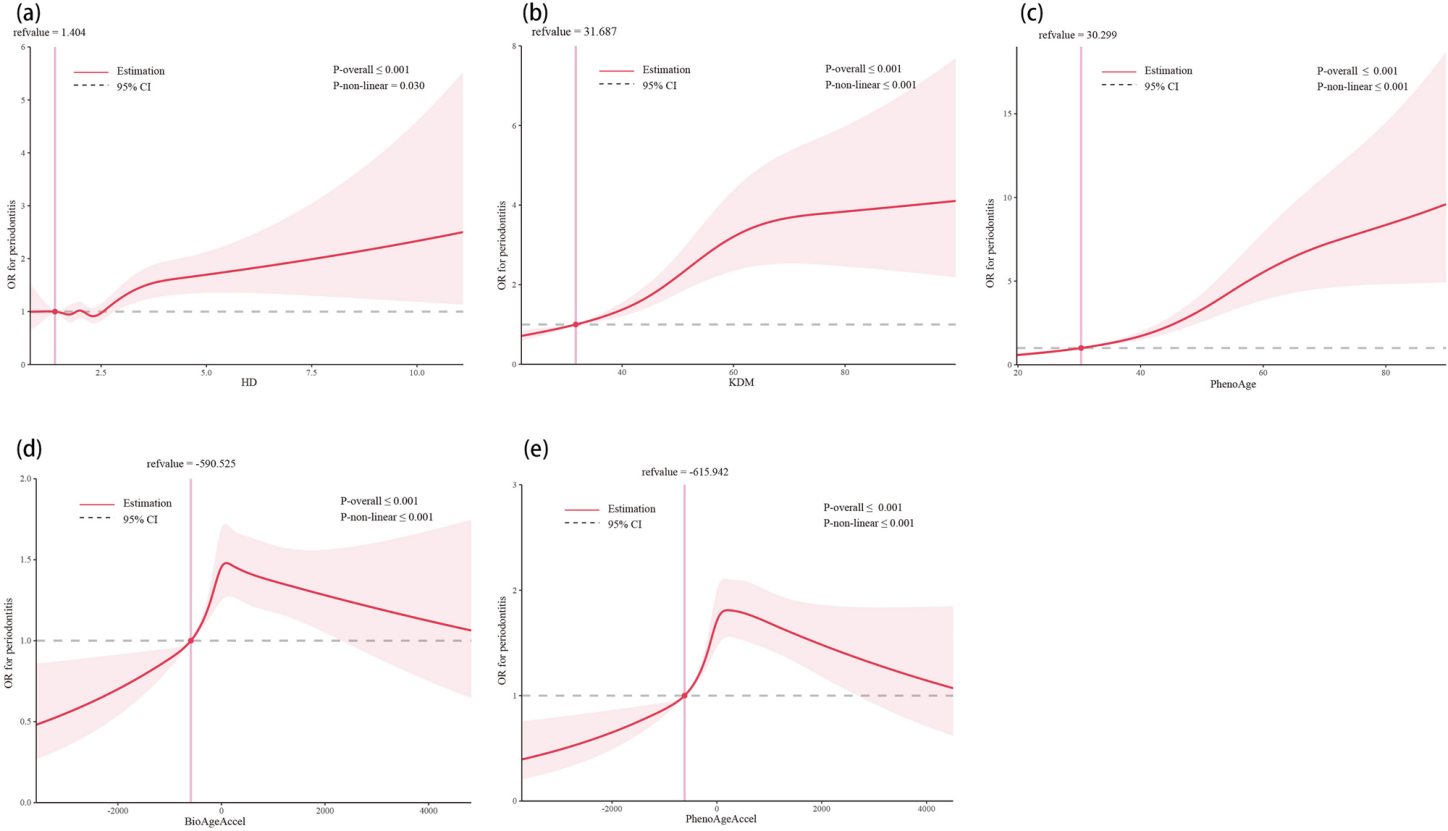
Restricted cubic spline model of the odds ratios of periodontitis with HD (**a**), KDM (**b**), PhenoAge (**c**), BioAgeAccel (**d**), and PhenoAgeAccel (**e**). All were adjusted for age, gender, race, educational level, marital status, ratio of family income to poverty, BMI, cigarette use, diabetes, arthritis, liver disease, and cancer. HD: homeostatic dysregulation; KDM: Klemera–Doubal method; OR: Odds ratio; CI: Confidence interval.

Figure 2 illustrates a significant non-linear positive relationship between the three biological ages and periodontitis (*P* for non-linearity < 0.001). The multivariate-adjusted odds ratio (OR) showed a short period of fluctuation followed by a rapid increase when the HD value exceeded 1.404. However, the risk tended to level off at or below this value. Regarding the S-shaped relationship between KDM and periodontitis, the plot revealed a slow increase in the risk of periodontitis within the lower range of KDM (< 31.687 years old). After reaching a KDM of 31.687 years old, the risk increased rapidly, but when KDM levels exceed approximately 70 years old, the risk of periodontitis enters a plateau period. Additionally, the risk of periodontitis remained relatively stable until around 30.299 years old of PhenoAge, after which it started to increase rapidly. The cutoff values for accelerated biological aging were -590.525 for BioAgeAccel and −615.942 for PhenoAgeAccel.

### MR analysis estimates of associations between accelerated biological aging and periodontitis

**Figure 3:**
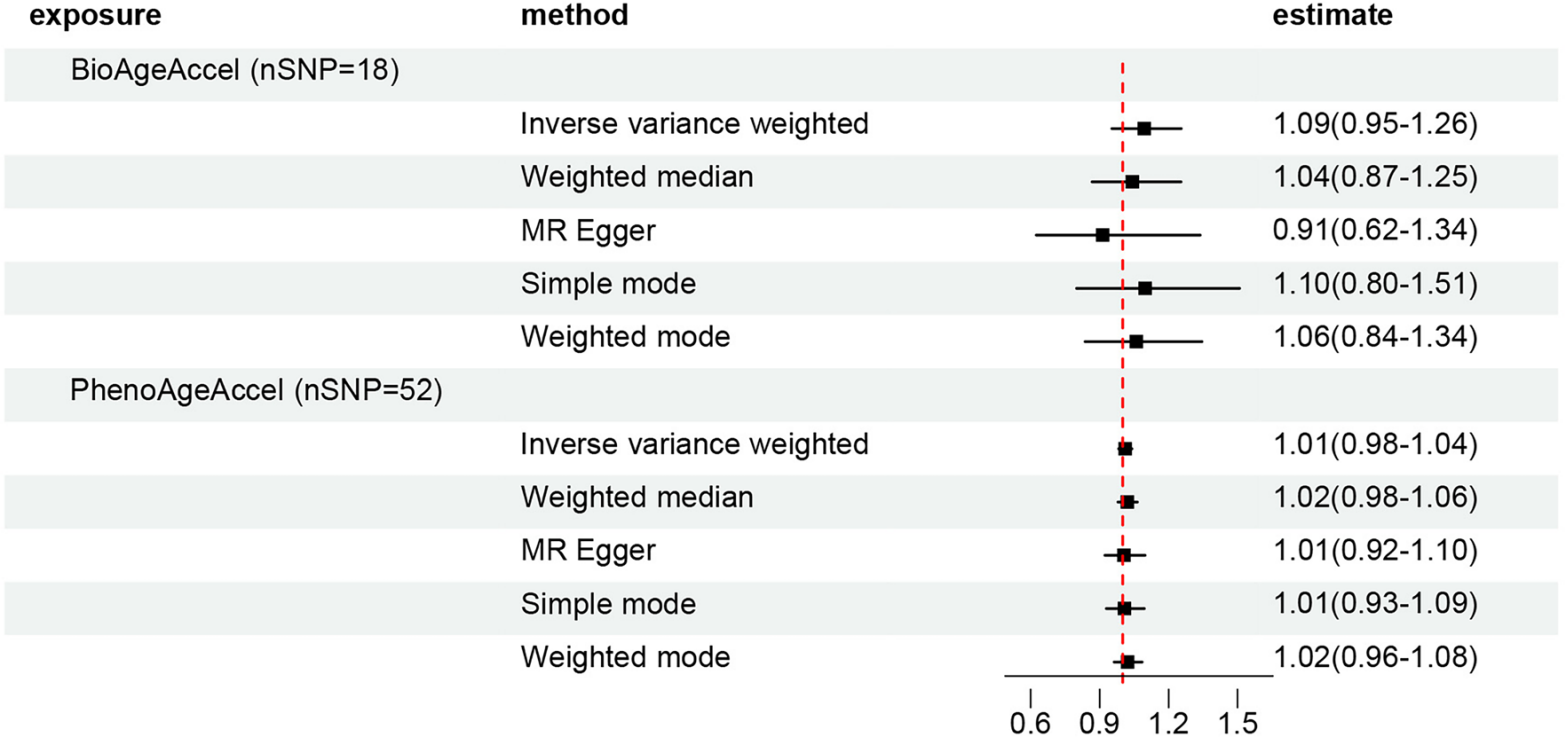
Forest plot of causal effects between accelerated biological aging and periodontitis. Mendelian randomization analyses results of Inverse variance weighted, Weighted median, MR Egger, Simple mode, and Weighted mode analyses.

For the MR analysis investigating the causal association of BioAgeAccel and PhenoAgeAccel with periodontitis, we obtained 18 (Supplementary Table S1) and 52 (Supplementary Table S2) instrumental variables, respectively. The results of the IVW regression indicated no causal effect of BioAgeAccel on periodontitis (OR 1.09, 95% CI 0.95–1.26, *p* = 0.20) (Fig. 3). Similar nonsignificant outcomes were observed in the analysis using the weighted median (OR 1.04, 95% CI 0.87–1.25, *p* = 0.66), MR Egger (OR 0.91, 95% CI 0.62–1.34, *p* = 0.65), simple mode (OR 1.20, 95% CI 0.80–1.51, *p* = 0.57), and weighted mode (OR 1.06, 95% CI 0.84–1.34, *p* = 0.63) methods. Similarly, no causal effects of PhenoAgeAccel on periodontitis were detected using IVW regression (OR 1.01, 95% CI 0.98–1.04, *p* = 0.46) or any other methods (Fig. 3, Supplementary Table S3).

### Sensitivity analysis

**Figure 4:**
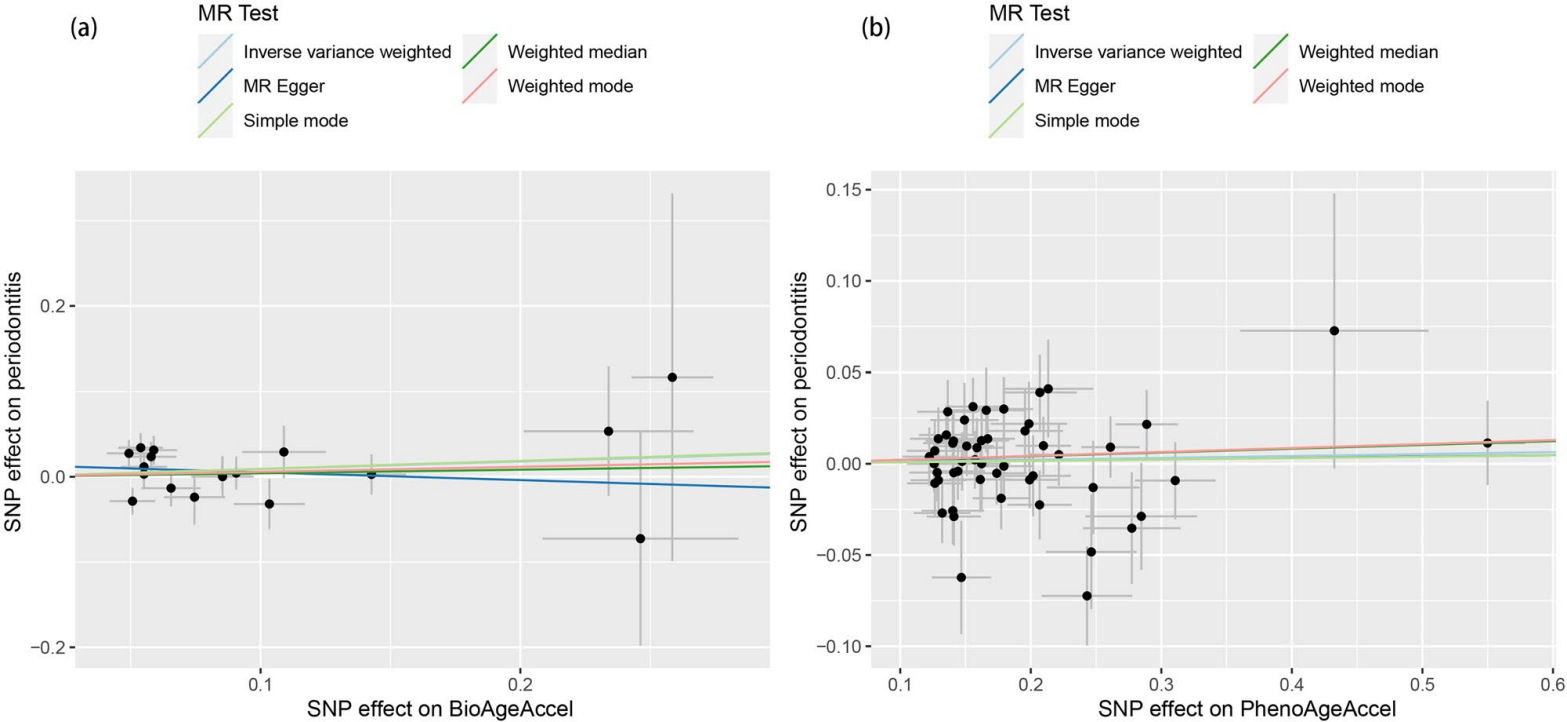
Scatter plots of the MR study investigating the effect of accelerated biological aging on periodontitis. (**a**) The effect of BioAgeAccel on periodontitis. (**b**) The effect of PhenoAgeAccel on periodontitis. MR: Mendelian randomization; SNP: single nucleotide polymorphism.

First, no significant statistical heterogeneity was found using the Cochran Q test (Supplementary Table S4, *p* = 0.35 for BioAgeAccel, *p* = 0.28 for PhenoAgeAccel). Second, to assess pleiotropy, we conducted the MR-Egger intercept test and the MR-PRESSO global test. The MR Egger intercept was not statistically different from zero when exposure was 0 (Fig. 4 and Supplementary Table S4), providing evidence of non-pleiotropy. The MR-PRESSO global test (all *p* > 0.05) also indicated non-pleiotropy when determining the pleiotropy significance for each SNP and the entire horizontal pleiotropy (Supplementary Table S4. Lastly, the leave-one-out analyses and symmetrical funnel plots (Supplementary Figure S1) did not identify any outlying individual instrumental variables, thus excluding the influence of outliers on our MR estimates.

## Discussion

Aging is a multifaceted biological phenomenon characterized by the deterioration and functional decline of various organs and tissues^33^. Periodontal disease, a prevalent oral pathology primarily initiated by bacterial infections, manifests with symptoms such as gum inflammation, bleeding, tooth mobility, and in extreme scenarios, may culminate in tooth loss^34^.

Emerging research underscores a profound association between aging and the susceptibility to periodontal disease^5^. The following physical or pathophysiological processes may be involved in and elucidates this relationship, encompassing both natural aging and accelerated aging processes. The first is the declination of the immune system^35^. As maturation progresses, there is a gradual waning of immune system efficacy, particularly pronounced in the elderly population. This immunosenescence renders older individuals more vulnerable to bacterial pathogens, notably those responsible for periodontal disease, thereby elevating their likelihood of developing such conditions. The second refers to chronic inflammation^36^. Concurrent with advancing age, there is an incremental augmentation in the body's propensity for inflammatory responses, potentially culminating in chronic inflammation. This persistent inflammatory state is intricately linked to the onset and progression of periodontal disease, as it can compromise the integrity and functionality of the periodontal structures. Then, as ageing, tissue repair capacity is gradually diminished. With advancing years, the inherent capacity for tissue regeneration and repair diminishes. Consequently, older individuals exhibit a slower recovery trajectory from periodontal insults, potentially exacerbating the severity of the condition. Besides, lifestyle factors take a part in the relationship between ageing and periodontal diseases^37^. Lifestyle determinants, including smoking, dietary habits, and oral hygiene practices, significantly modulate the interplay between aging and periodontal disease. While smoking and suboptimal diets amplify the risk of periodontal disease, diligent oral hygiene can mitigate its incidence. Investigations into accelerated aging have unveiled a myriad of molecular and cellular mechanisms that are implicated in the development of periodontal disease. Oxidative stress and DNA damage, for instance, are pivotal factors in accelerated aging and share a significant correlation with the etiopathogenesis of periodontal disease^38^. Taken together, a robust correlation exists between aging, the acceleration thereof, and the predilection for periodontal disease. Furthermore, unraveling the mechanisms underlying accelerated aging may provide critical insights that could inform the innovation of novel therapeutic strategies for periodontal disease.

This study represents the first investigation reporting the relationship between biological aging and periodontitis, utilizing a large-scale survey data from NHANES 2009–2014 and MR analysis. The measurement of biological aging was divided into two components: biological age and accelerated biological aging. Biological ages, namely HD, KDM, and PhenoAge, were calculated based on a series of clinical biochemical markers. BioAgeAccel and PhenoAgeAccel, calculated from the residual between KDM and PhenoAge, were used to measure the rate of aging. Both biological ages and accelerated aging were found to be associated with periodontitis. These associations were non-linear and independent of chronological age, demographic factors, and disease conditions. However, MR analysis did not provide evidence of a causal relationship between accelerated aging and periodontitis.

This large-scale cross-sectional study aligns with previous mechanistic studies supporting the associations of clinical biochemical biomarkers related to biological aging with periodontal disease. The aging process promotes pathogenic microbial colonization and impairs the vitality of mesenchymal stromal stem cells, leading to a pro-inflammatory microenvironment that exacerbates inflammatory infiltrates and bone loss in the periodontium^39,40^. Several inflammatory factors are relevant to the periodontitis, such as TNF-α, IL-1β, and IL-6^41^. Moreover, studies have shown that aging is associated with a decline in alkaline phosphatase activity in the periodontal ligament, resulting in calcification degeneration and degradation of the periodontium^42^. Prevention strategies that regulate the activity of alkaline phosphatase may make an effect on periodontitis^41,43^. A study categorized individuals undergoing maintenance hemodialysis based on their periodontal status and compared the levels of clinical parameters between groups. The findings revealed that high-sensitivity C-reactive protein, blood urea nitrogen, creatinine, transferrin, erythropoietin, and albumin are associated with poor periodontal status^44^. Furthermore, a set of 19 blood biomarker signatures, including constituents of standard hematological measures, lipid biomarkers, and markers of inflammation and frailty, have been correlated with longitudinal changes in physiological functions and the risk of cancer, cardiovascular disease, type 2 diabetes, and mortality^45^. However, the absence of suitable models assessing biological ages makes it difficult to explore the relationship between biological aging measured from clinical biochemical biomarkers and periodontitis. Our study addresses this gap by utilizing a modern toolkit for precise biological age calculation.

In this study, since we have not yet confirmed the authentic connection between periodontitis and aging, we utilized logistic regression, a general linearity assess model, to assume and detect whether the two variables underwent a linear relationship between biological aging and periodontitis at first. However, this relatively linear assumption may result in information loss and may not accurately reflect reality^46^. To address this issue, restricted cubic spline regression was utilized. As a potential alternative to logistic regression, restricted cubic spline regression does not assume linearity and provides a visual representation of the nonlinear relationship. In the analysis of the three biological ages and two accelerated biological aging measures, all p-values for nonlinearity were less than 0.05, indicating a nonlinear relationship between biological aging and periodontitis. Additionally, we observed an interesting trend where the risk of periodontitis appeared to increase rapidly after KDM or PhenoAge reached 31.687 or 30.299 years, respectively (Fig. 2). These cutoff points for active periodontitis were close to the age of 30, a point at which previous studies have indicated a high prevalence of chronic periodontal disease^47^.

The main strength of this study lies in its large sample size, derived from the NHANES and GWAS databases. The use of logistic regression and restricted cubic spline regression facilitated the exploration of both linear and nonlinear relationships, respectively, and allowed for the calculation of cutoff points. Adjustment for covariates helped to mitigate the influence of confounding factors on the main conclusions. Additionally, to the best of our knowledge, this represents the first study to simultaneously assess and investigate biological ages and the rate of biological aging in relation to the periodontitis. Previous observational studies had not examined the association between periodontitis and three biological ages. Likewise, no MR studies have investigated the causal relationship between biological aging and periodontitis. This may be attributed to the fact that representative methods for calculating biological age have only recently been introduced^19^. Besides, performing extensive large-scale trial data collection to thoroughly clarify these causal relationships encounters ethical and moral constraints. Therefore, it is more practical to gather evidence using MR methods^48^. Our current MR study has addressed the limitations inherent in observational research by establishing a non-causal association between accelerated biological aging and periodontitis, thereby mitigating confounding factors and minimizing the potential for reverse causation biases.

However, several limitations should be noted in this study. Firstly, the negative causal relationship identified through the MR study warrants further investigation. While our observational data suggest a potential non-linear association between biological aging and periodontitis, the MR study exclusively examined a linear causal relationship, so non-linear causality cannot be excluded. Additionally, the NHANES study included participants from the US, while the individuals analyzed in the MR study were European. The discrepancy in ancestry may contribute to the contrast between the observed relativity and the absence of a causal association. Consistent with our findings, previous combined association-causality studies have also tended to attribute inconsistencies in results to population mismatches^49,50^. Secondly, only the data of accelerated biological aging are available in GWAS database, that contributes to the results acquirement, while no specific GWAS data reflecting the other three biological ages, which were analyzed in our cross-sectional study, are currently available for MR analyses. Therefore, we were only able to explore the relativity between the three biological ages and periodontitis, without establishing a causal association. Besides, comorbidity is a vital confounder during aging. Comorbidity refers to the condition where a patient simultaneously suffers from other diseases. Research indicates that the presence of comorbidities may intensify the severity of periodontitis and affect the efficacy of treatment. For instance, diabetic patients are prone to exacerbate oral bacterial infections due to poor blood sugar control, which can exacerbate periodontal disease^51,52^. Besides, the diabetic microenvironment, such as elevated levels of glucose and lipids, can contribute to an increased burden of senescent cells, thereby accelerating the aging process^53^. Cardiovascular disease patients may experience issues such as hypoxia and malnutrition in periodontal tissues due to compromised blood circulation^54^. Hence, comorbidity is an important confounding factor in this study. We have meticulously selected from databases and carefully reviewed subjects' medical histories to exclude samples with severe chronic diseases, aiming to minimize their impact on the results and enhance the reliability of our findings. The presence of comorbidities can worsen the severity of periodontitis and influence the therapeutic outcomes. Therefore, age and comorbidity status should be taken into account when preventing and treating periodontitis. Future research could further investigate other potential effects of comorbidities on the relationship between periodontitis and aging. Finally, the measurement of biological aging is still a work in progress. It is necessary to quantify the aging process using ongoing techniques and examine its associations with periodontitis.

## Conclusion

In conclusion, this study revealed nonlinear associations between biological aging, as well as accelerated aging, and periodontitis, using large-scale survey data and MR analysis. Although observational evidence suggested a correlation between higher biological ages and an increased risk of periodontitis, the MR study did not establish causality. These findings emphasize the significance of considering biological aging in understanding periodontitis and call for further research to elucidate underlying mechanisms and interventions to give implications in clinical translation.

### Supplementary Information

Supplementary Information 1.

Supplementary Information 2.

Supplementary Information 3.

Supplementary Information 4.

Supplementary Information 5.

Supplementary Information 6.

Supplementary Information 7.

## Data Availability

The datasets of NHANES and MR analysis used in this study can be found in [NHANES datasets] https://wwwn.cdc.gov/nchs/nhanes; [MR analysis datasets for Exposure (BioAgeAccel and PhenoAgeAccel) and Outcome (periodontitis)] https://figshare.com/articles/dataset/BioAgeAccel_GWAS_summary_statistics/12620366/1; https://figshare.com/articles/dataset/PhenoAgeAccel_GWAS_summary_statistics/12620291/1; https://data.bris.ac.uk/data/dataset/2j2rqgzedxlq02oqbb4vmycnc2.

## Abbreviations

KDM: Klemera–Doubal method
HD: Homeostatic dysregulation
CHANGES: National Health and Nutrition Examination Survey
MR: Mendelian randomization
PD: Probing depth
AL: Attachment loss
BMI: Body mass index
GWAS: Genome-wide association study
IVW: Inverse-variance-weighted
OR: Odds ratio
CI: Confidence interval
