# Supplementary material for: Association between biological aging and periodontitis using NHANES 2009–2014 and mendelian randomization
Source: Sci Rep. 2024 May 2;14:10089. doi: 10.1038/s41598-024-61002-9 (PMC11065868; doi:10.1038/s41598-024-61002-9)
Supplement: Supplementary file 1 — Supplementary Information 1. [file 41598_2024_61002_MOESM1_ESM.docx]

**Supplementary Figure 1 MR leave‐one‐out and funnel plots sensitivity analysis for accelerated biological aging on periodontitis**

**
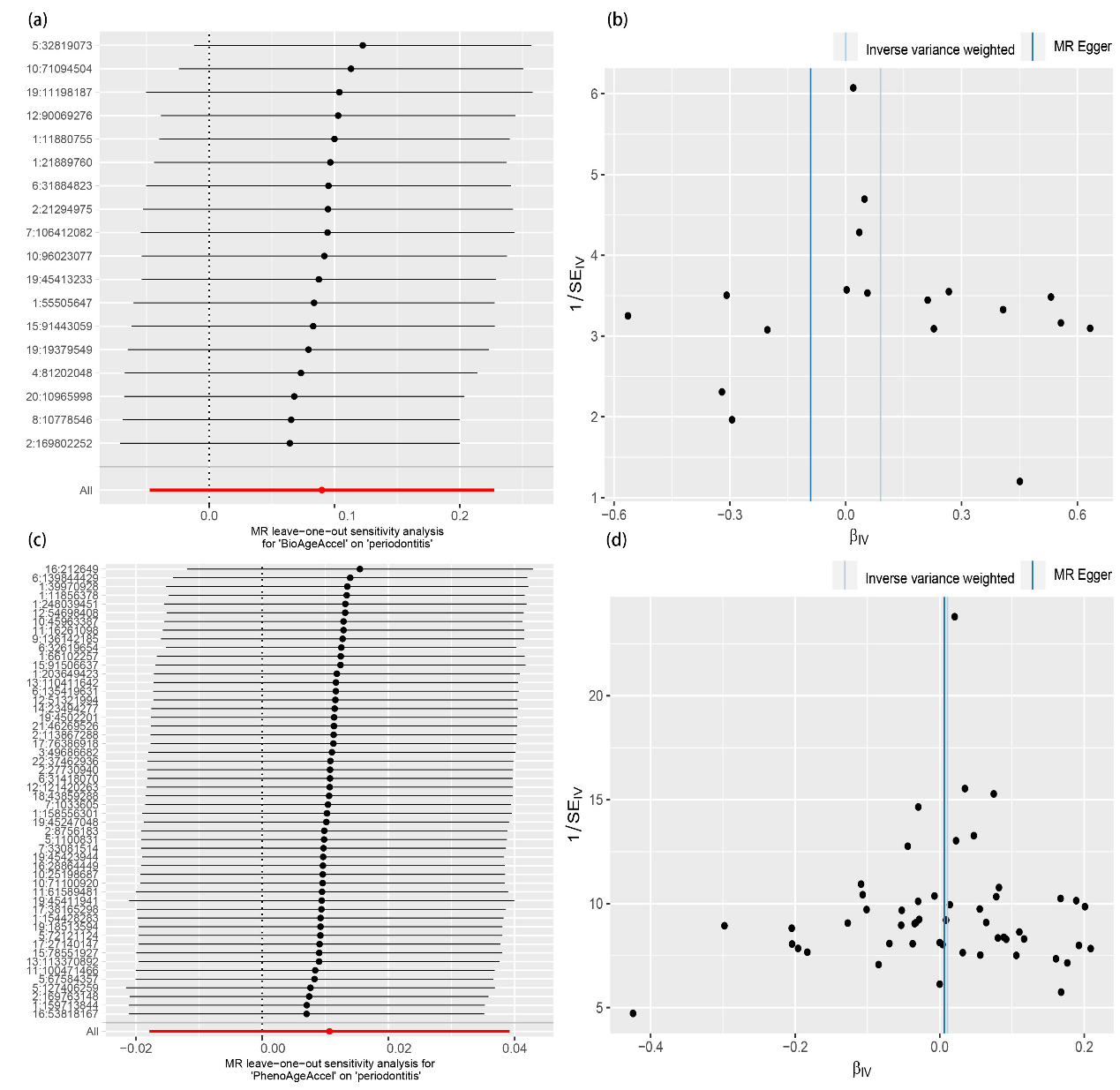
**

Supplementary Figure 1. MR leave‐one‐out and funnel plots sensitivity analysis for accelerated biological aging on periodontitis. (a), (b) Leave‐one‐out and funnel plots sensitivity analysis for BioAgeAccel on periodontitis. (c), (d) Leave‐one‐out and funnel plots sensitivity analysis for PhenoAgeAccel on periodontitis.
