# Supplementary material for: Association between biological aging and periodontitis using NHANES 2009–2014 and mendelian randomization
Source: Sci Rep. 2024 May 2;14:10089. doi: 10.1038/s41598-024-61002-9 (PMC11065868; doi:10.1038/s41598-024-61002-9)
Supplement: Supplementary file 4 — Supplementary Information 4. [file 41598_2024_61002_MOESM4_ESM.docx]

Supplementary Table 3 Causal effects of accelerated biological aging on periodontitis

| Exposure | Method | Number of SNPs | Beta | SE | P | OR (95% CI) |
| --- | --- | --- | --- | --- | --- | --- |
| BioAgeAccel | Inverse variance weighted | 18 | 0.090 | 0.070 | 0.200 | 1.09(0.95, 1.26) |
|  | Weighted median | 18 | 0.042 | 0.094 | 0.659 | 1.04(0.87, 1.25) |
|  | MR Egger | 18 | -0.091 | 0.195 | 0.647 | 0.91(0.62, 1.34) |
|  | Simple mode | 18 | 0.094 | 0.162 | 0.571 | 1.10(0.80, 1.51) |
|  | Weighted mode | 18 | 0.059 | 0.121 | 0.635 | 1.06(0.84, 1.34) |
| PhenoAgeAccel | Inverse variance weighted | 52 | 0.011 | 0.015 | 0.464 | 1.01(0.98, 1.04) |
|  | Weighted median | 52 | 0.021 | 0.021 | 0.327 | 1.02(0.98, 1.06) |
|  | MR Egger | 52 | 0.006 | 0.044 | 0.885 | 1.01(0.92, 1.10) |
|  | Simple mode | 52 | 0.008 | 0.042 | 0.85 | 1.01(0.93, 1.09) |
|  | Weighted mode | 52 | 0.022 | 0.030 | 0.479 | 1.02(0.96, 1.08) |

Notes: OR: Odds ratio, SE: Standard error, CI: Confidence interval
