# Supplementary material for: Association between biological aging and periodontitis using NHANES 2009–2014 and mendelian randomization
Source: Sci Rep. 2024 May 2;14:10089. doi: 10.1038/s41598-024-61002-9 (PMC11065868; doi:10.1038/s41598-024-61002-9)
Supplement: Supplementary file 5 — Supplementary Information 5. [file 41598_2024_61002_MOESM5_ESM.docx]

Supplementary Table 4 Sensitivity analysis of the causal relationship between accelerated biological aging and periodontitis

| Sensitivity analysis | | BioAgeAccel | PhenoAgeAccel |
| --- | --- | --- | --- |
| Cochran Q test | Q value | 18.577 | 56.118 |
|  | P | 0.353 | 0.289 |
| MR Egger | Intercept | 0.014 | 0.001 |
|  | P | 0.334 | 0.919 |
| MR PRESSO | P | 0.275 | 0.226 |
