# Supplementary material for: Association between biological aging and periodontitis using NHANES 2009–2014 and mendelian randomization
Source: Sci Rep. 2024 May 2;14:10089. doi: 10.1038/s41598-024-61002-9 (PMC11065868; doi:10.1038/s41598-024-61002-9)
Supplement: Supplementary file 7 — Supplementary Information 7. [file 41598_2024_61002_MOESM7_ESM.docx]

| **Supplementary Table 6. Sensitive analysis of the cross-sectional study**  Supplementary Table 6.1 Sensitive analysis of biological age with mean PD | | | | | | |  |
| --- | --- | --- | --- | --- | --- | --- | --- |
| Biological Aging | Model 1 | | Model 2 | | Model 3 | |  |
|  | β (95% CI) | p | β (95% CI) | p | β (95% CI) | p | |
| HD | 0.069(0.048,0.091) | <0.001 | 0.036(0.015,0.057) | 0.001 | 0.038(0.016,0.059) | <0.001 | |
| KDM | 0.012(0.009,0.015) | <0.001 | 0.009(0.006,0.012) | <0.001 | 0.009(0.006,0.012) | <0.001 | |
| PhenoAge | 0.022(0.018,0.026) | <0.001 | 0.014(0.010,0.019) | <0.001 | 0.015(0.011,0.020) | <0.001 | |
| BioAgeAccel | 0.100(0.068,0.133) | <0.001 | 0.066(0.035, 0.096) | <0.001 | 0.066(0.036,0.096) | <0.001 | |
| PhenoAgeAccel | 0.163(0.121,0.204) | <0.001 | 0.095(0.054,0.137) | <0.001 | 0.097(0.054,0.139) | <0.001 | |
| Notes: Model 1: Adjusting for age, gender, race;  Model 2: Model 1 + adjusted for educational level, marital status, ratio of family income to poverty, BMI, and cigarette use.  Model 3: Model 2 + adjusted for diabetes, arthritis, liver disease, and cancer.  PD: probing depth; HD: homeostatic dysregulation; KDM: Klemera–Doubal method;  CI: Confidence interval; | | | | | | | |

| Supplementary Table 6.2 Sensitive analysis of biological age with mean CAL | | | | | | |  |
| --- | --- | --- | --- | --- | --- | --- | --- |
| Biological Aging | Model 1 | | Model 2 | | Model 3 | |  |
|  | β (95% CI) | p | β (95% CI) | p | β (95% CI) | p | |
| HD | 0.168(0.134,0.203) | <0.001 | 0.102(0.075,0.129) | <0.001 | 0.095(0.065,0.126) | <0.001 | |
| KDM | 0.020(0.015,0.025) | <0.001 | 0.015(0.011,0.020) | <0.001 | 0.014(0.010,0.019) | <0.001 | |
| PhenoAge | 0.041(0.036,0.046) | <0.001 | 0.028(0.023,0.033) | <0.001 | 0.028(0.024,0.032) | <0.001 | |
| BioAgeAccel | 0.135(0.084,0.185) | <0.001 | 0.082(0.037, 0.128) | <0.001 | 0.075(0.029,0.121) | 0.002 | |
| PhenoAgeAccel | 0.310(0.251,0.368) | <0.001 | 0.201(0.152,0.250) | <0.001 | 0.193(0.143,0.242) | <0.001 | |
| Notes: Model 1: Adjusting for age, gender, race;  Model 2: Model 1 + adjusted for educational level, marital status, ratio of family income to poverty, BMI, and cigarette use.  Model 3: Model 2 + adjusted for diabetes, arthritis, liver disease, and cancer.  CAL: attachment loss; HD: homeostatic dysregulation; KDM: Klemera–Doubal method; CI: Confidence interval; | | | | | | | |
